# HTRgene: a computational method to perform the integrated analysis of multiple heterogeneous time-series data: case analysis of cold and heat stress response signaling genes in Arabidopsis

**DOI:** 10.1186/s12859-019-3072-2

**Published:** 2019-12-02

**Authors:** Hongryul Ahn, Inuk Jung, Heejoon Chae, Dongwon Kang, Woosuk Jung, Sun Kim

**Affiliations:** 1 Department of Computer Science and Engineering, Seoul National University, Seoul, Korea; 2 Department of Computer Science and Engineering, Kyungpook National University, Daegu, Korea; 3 Division of Computer Science, Sookmyung Women’s University, Seoul, Korea; 4 Department of Crop Science, Konkuk University, Seoul, Korea; 5 Interdisciplinary Program in Bioinformatics, Seoul National University, Seoul, Korea; 6 Bioinformatics Institute, Seoul National University, Seoul, Korea

**Keywords:** Integration analysis, Multiple time-series gene expression data, Stress response, Response order preserving DEG

## Abstract

**Background:**

Integrated analysis that uses multiple sample gene expression data measured under the same stress can detect stress response genes more accurately than analysis of individual sample data. However, the integrated analysis is challenging since experimental conditions (strength of stress and the number of time points) are heterogeneous across multiple samples.

**Results:**

**HTRgene** is a computational method to perform the integrated analysis of multiple heterogeneous time-series data measured under the same stress condition. The goal of HTRgene is to identify “response order preserving DEGs” that are defined as genes not only which are differentially expressed but also whose response order is preserved across multiple samples. The utility of HTRgene was demonstrated using 28 and 24 time-series sample gene expression data measured under cold and heat stress in Arabidopsis. HTRgene analysis successfully reproduced known biological mechanisms of cold and heat stress in Arabidopsis. Also, HTRgene showed higher accuracy in detecting the documented stress response genes than existing tools.

**Conclusions:**

HTRgene, a method to find the ordering of response time of genes that are commonly observed among multiple time-series samples, successfully integrated multiple heterogeneous time-series gene expression datasets. It can be applied to many research problems related to the integration of time series data analysis.

## Introduction

Over the past two decades, the rapid development of molecular measurement technologies, such as microarray [1] and RNA sequencing (RNA-Seq) [2], have improved scalability and accuracy and reduced time and cost in measuring expression levels of all genes in a cell, which is known as transcriptome data. Analyzing transcriptome data can be very helpful in understanding complex biological mechanisms. Among many research questions, understanding how plants respond to environmental stress such as drought, salt, cold and heat is an important research problem. Then, using large-scale parallel measurement techniques, transcriptome data are measured under stress conditions to identifying stress response genes.

Analysis of detecting differentially expressed genes (DEGs) has been widely performed [3] to identify stress response signaling genes from transcriptome data that are measured under stress condition. However, detecting DEGs in different samples showed discordant results even though the experiments were conducted with the same stimulus on the same species. For example, Kreps [3] and Matsui [4] reported 2086 and 996 DEGs for cold stress in Arabidopsis, respectively, and only 232 DEGs, about 16% of the union of two DEG sets, were commonly determined. This result shows the requirement of a robust analysis of gene expression datasets.

### Motivation and related works

The motivation of this paper is to propose a more robust DEG detection method by integrated analysis of multiple gene expression data of a stress. The integrated analysis for DEG detection is now possible since time-series gene expression datasets measured under the same stress are increasing and they are available for integrated analysis. For instance, the OryzaExpress database [5] provides 624 gene expression datasets from 37 experimental series with their experimental conditions. Its improved version, PlantExpress [6] provides microarray gene expression data of 3884 and 10,940 samples for rice and Arabidopsis species, and the Rice Expression Database (RED) [7] provides 284 RNA-seq gene expression data that were measured under various experimental conditions in rice species.

The integrated analysis for DEG detection will be a new type of approach of DEG detection because there are many DEG methods so far but existing methods mainly focused on individual experimental analysis and did not consider the interrelationships with other samples. For instance, the pair-wise DEG detection approach that compares the expression value of gene before and after stress treatment using statistical models, such as DESeq [8], edgeR [9], and limma [10] and the time-series DEG detection approach that considers time domain information, such as maSigPro [11], Imms [12], splineTC [13], and ImpulseDE [14] did not consider multiple sample analysis. We expect that integrated analysis will provide robust DEG results since it is well known that when more data is used for the analysis, the signal to noise becomes clearer and the accuracy of the results improves.

### Challenges and our approach

Heterogeneous meta-properties [15, 16] is a challenge for the integrated analysis of multiple time-series gene expression datasets. Meta-property is external information of data that is related to the experimental design and condition, e.g., tissue of samples, age of samples, time points, and so forth. When we collected the multiple time-series data from the gene expression database, the meta-properties are usually heterogeneous since they are independently created by different research groups. For instance, suppose that two datasets of heat stress experiments were generated with different meta-properties: 14 days old, 43 ^∘^C heat stress, <0,2,8> hours vs. 21 days old, 38 ^∘^C heat stress, <0,2,4,10> hours.

Generally, DEG detection analysis of stress data investigates the change of gene expression levels before and after the response time to the stress. However, heterogeneous meta-properties cause the difficulty to specify the response time.

- Different environmental conditions cause the difference in the biological system’s response timing to stress. For example, the response time of the same gene is delayed in stress-resistant condition sample (e.g. 4h in mature and low temperature-treated sample) relative to stress-sensitive condition sample (e.g. 2h in infant and high temperature-treated sample).
- Different time points cause unmeasured time points in the time series dataset. Therefore, we may not know the expression levels in another sample data.

The unspecified response time issue makes the integrated analysis of time-series data much more challenging than analysis of an individual time-series data. In order to address the unspecified response time issue, our work is based on an idea that *the response order of genes will be preserved* even if the response time of genes is delayed or advanced across multiple samples. It is based on the biological knowledge that biological adaptation to stress is a deterministic and sequential process; a gene activates the target genes and this regulation continues according to a deterministic stress response pathway. Based on this idea, we developed HTRgene, a method to identify “response order preserving DEGs” for multiple time-series samples.

## Methods

### HTRgene algorithm

HTRgene is an algorithm to identify “response order preserving DEGs” by the integrated analysis of multiple heterogeneous time-series gene expression datasets. To define “response order preserving DEGs”, *stress response time* is defined based on a study of Chechik and Yosef [17, 18]. They reported when a cell is exposed under stress, the expression level of a gene increases or decreases at a certain time point and remains stable. Thus, we defined the response time point of a gene as a time point at which the expression level of the gene statistically changes before and after the time point. Then, “Response order preserving DEGs” are defined as genes not only which are differentially expressed but also whose response order is preserved across multiple samples. Below are the detailed definitions of response time and response order preserving DEGs.

#### **Definition 1**

Suppose that time-series sample *i* is measured at *l*_*i*_ time points, resulting in *e*_*g*,*i*,*j*_, the expression level of a gene *g* in sample *i* at time point *j*. Then, let *A*_*g*,*i*,*j*_ be a set of expression levels of a gene *g* in sample *i* after time point *j* including *j*, i.e., $\{e_{g,i,j}, \dots, e_{g,i,l_{i}}\}$. Let also *B*_*g*,*i*,*j*_ be a set of expression levels of a gene *g* in sample *i* before time point *j* excluding *j*, i.e., {*e*_*g*,*i*,1_,…,*e*_*g*,*i*,*j*−1_}.

**A response time (RT)**, $t_{g}^{i}$, is a time point of a gene *g* in sample *i* where a statistical test of significance of expression level difference is maximized between $B_{g,i,t_{g}^{i}}$ and $A_{g,i,t_{g}^{i}}$. **A response time vector**, $\vec {R_{g}}$, is a vector of response times of a gene *g* for *m* samples, i.e., $< t_{g}^{1}, \dots, t_{g}^{m}>$. **The order of two response time vectors**$\vec {R_{g_{1}}}$ and $\vec {R_{g_{2}}}$ is determined as $\vec {R_{g_{1}}} \preceq \vec {R_{g_{2}}}$ if $t_{g_{1}}^{^{\bullet }} < t_{g_{2}}^{^{\bullet }}$ for at least one sample and $t_{g_{1}}^{^{\bullet }} \leq t_{g_{2}}^{^{\bullet }}$ for all samples. **A longest response schedule** is a longest consistent ordering of genes for a set of binary ordering of two genes based on response time vectors.**Response order preserving DEGs** are defined as DEGs belonging to the longest response schedule.**A response phase** is the position of response in the response schedule.

Below introduce two computational issues in discovering response order preserving DEGAS.

- **Complexity issue:** The number of genes determines the complexity of determining and ordering response times. It is known that 27,416 coding genes exist in Arabidopsis [19], which results in very high complexity.
- **Noise issue:** Noise often occurs when measuring gene expression. The noise of the expression value of a gene can cause the noise of response time followed by the entire response ordering, resulting in the overall result unstable.

**Fig. 1:**
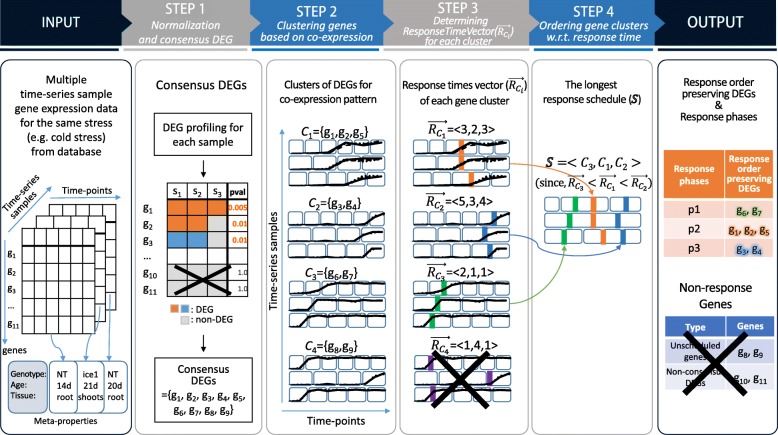
Overview of the HTRgene algorithm. The input of HTRgene is a set of multiple time-series gene expression data of the same stress (e.g cold stress) that is collected from databases. Step 1 normalizes the data and detects consensus DEGs. Step 2 partitions the consensus DEGs into gene clusters with high co-expression patterns. Step 3 determines a response time vector $\vec {R_{C_{i}}}$ for each gene cluster. Step 4 orders gene clusters based on their response time. The final output of HTRgene, response order preserving DEGs and their response phases, are produced

HTRgene’s idea to reduce complexity and noise effect is to determine and order the response times at the gene cluster level, not at the gene level. Figure 1 showed the four step workflow of HTRgene: 1) selecting consensus DEGs (i.e., genes that are differentially expressed in common across multiple time-series samples), 2) clustering the DEGs based on the co-expression pattern, 3) detecting the response times for each gene cluster, 4) ordering the clusters according to the response times, resulting in “response order preserving DEGs.”

### Step 1: Normalization and detection of consensus DEGs

The input of HTRgene is a set of time-series gene expression data from a single platform, either microarray or RNA-Seq. Scale normalization methods are used depending on the data platform. Quantile normalization using the affy R package [20] is used for microarray data, and variance stabilization transformation using the DESeq package [8] is used for RNA-Seq data. After scale normalization, HTRgene performs base normalization to set the expression value at the initial time point (*T*=0) to zero. Different base normalization methods are used depending on the shape of data distribution. For instance, when plotting expression levels of a gene, the plot follows a normal distribution, so substitution-based normalization (Eq. 1) is used for normal-shaped data. However, log-fold-change-based normalization (Eq. 2) is used for log-scale-shape distribution data, which is the standard practice for RNA-Seq data.

The expression level *e*_*g*,*i*,*j*,*k*_ of gene *g* measured in time-series sample *i* at time point *j* in a replicate *k* is adjusted as follows for microarray data:

1 $$ e_{g,i,j,k} - \frac{1}{|R|}\sum\limits_{k}^{|R|} e_{g,i,0,k} \text{,}  $$

and as follows for RNA-Seq data:

2 $$ log (e_{g,i,j,k}+1) - \frac{1}{|R|}\sum\limits_{k}^{|R|} log(e_{g,i,0,k}+1) \text{.}  $$

From normalized time-series gene expression data, HTRgene discovers consensus DEGs that are differentially expressed across multiple time-series samples. First, differential expression tests are performed using the limma [10] tool for each time point against the initial time point (*T*=0). If a gene is differentially expressed in at least one time domain in the sample, the gene is considered a DEG in a single time-series sample. After detecting single sample DEGs for each sample, a gene × sample matrix is constructed, where the (*i*,*j*) element is 1 if gene *i* are determined as a DEG in sample *j* or 0 otherwise.

Then, a statistical test is performed to investigate the number of samples in which a gene could be a consensus DEG for multiple samples. The elements of the gene × sample matrix are randomly shuffled, and how many samples contain DEGs is counted to generate a background distribution of DEG frequency. Then, the *p*-value of DEG frequencies is measured, and Benjamini-Hochberg multiple correction [21] is performed. Then, the genes whose DEG frequencies are significant (*adj*.*p*<0.05) are considered consensus DEGs.

### Step 2: Co-expression-based clustering of genes

To determine the response time points of the multiple time-series samples, clustering of genes is performed across different samples. To address a three dimension issue of multiple time-series samples (genes × samples × time points), our clustering analysis considers an approach that TimesVetor [22] proposed. The expression values of the time and the sample dimensions are concatenated to generate a single vector for each gene. Then, clustering analysis is performed for the gene expression vectors using the cosine distance and the skmeans [23] method. In this way, *K* gene clusters are produced, {*C*_1_,…,*C*_*K*_}. Among them, small-sized clusters with less than three member genes are discarded.

### Step 3: Detection of response time for each gene cluster

The goal of this step is to determine the response time vector $\vec {R_{C_{i}}}$ for each gene cluster *C*_*i*_. Determining an optimal response time vector is a computationally complex problem because of its exponentially increased search space. To handle the big search space issue, a hill-climbing approach is used to determine the optimal RT solution suggested in [24]: 1) an RT is initialized, 2) candidates of RT are generated, and 3) a new RT is selected that improves the separation score. Repeating substeps 2 and 3 are terminated when no candidate RTs improve the separation score.

#### Initializing $\vec {R_{C_{i}}}$ using a hierarchical clustering

The hierarchical clustering of genes is used to generate the initial $\vec {R_{C_{i}}}$. Since the goal is to determine a time point as a stress response time, hierarchical clustering is performed on the time dimension, progressively merging adjacent time points based on gene expression values. To set the initial $\vec {R_{C_{i}}}$, a response time *r*_*i*_ is determined for each sample *i* for all genes in *C*_*i*_ and then $\vec {R_{C_{i}}}$ is a vector $< t^{1}_{C_{i}}, \dots, t^{s}_{C_{i}}, \dots, t^{m}_{C_{i}}>$ where $t^{s}_{C_{i}}$ is a response time for each sample *s*. For convenience, we will omit *C*_*i*_ when we discuss an RT.

#### Generating and selecting a new candidate $\vec {R_{C_{i}}}$

After initialization of a RT, candidates of $\vec {R}$ are generated by moving an element of $\vec {R}$ to a nearby time point. Then, the quality score of $\vec {R}$ for each candidate $\vec {R}$ is computed by performing a t-test on the gene expression difference before and after a $\vec {R}$ vector as follows.

Let $EXP^{pre}_{g_{j}}$ and $EXP^{post}_{g_{j}}$ be sets of expression values of gene *g*_*j*_∈*C*_*i*_. The expression values of gene *g*_*j*_ of sample *s*_*i*_ before the response time point are assigned to $EXP^{pre}_{g_{j}}$, and the expression values after the response point are assigned to $EXP^{post}_{g_{j}}$. Then, ${Tstat}^{\vec {R_{g_{j}}}}$ is defined as the absolute value of t-statistics with an assumption of two-sample equal variance. Then, ${Tstat}^{\vec {R_{C_{i}}}}$, the quality score of a cluster *C*_*i*_, is defined as an average of quality scores of all genes in *C*_*i*_.

After measuring ${Tstat}^{\vec {R_{C_{i}}}}$ for each candidate RT vector $\vec {R}$, an RT that improves the separation score most is selected as a new RT.

### Step 4: Ordering of gene clusters according to response time

Among all clusters, the goal is to select and order a set of clusters that are consistent in terms of response times. To do this, the concept of *ResponseSchedule* is defined. Informally, a response schedule $\mathbb {S}$ is the most extended consistent sequence of response time vectors without any conflict. Here, "conflict" means that the order between two response time vectors can not be determined. For instance, two response time vectors <1,2,3,4> and <1,3,2,4> conflict since the second and third elements have disagreeing orders.

In this study, $\mathbb {S}$ is extended using a greedy search strategy. $\mathbb {S}$ is initialized to an empty set, and each cluster is considered in the order of quality scores ${Tstat^{R}}_{C_{\bullet }}$. The cluster with the highest quality score is added to $\mathbb {S}$. Then, the cluster *C*_*i*_ with the next best quality score is tested whether *C*_*i*_ has conflicts with any of the clusters that are already included in $\mathbb {S}$ or not. If so, *C*_*i*_ is added to $\mathbb {S}$, otherwise, *C*_*i*_ is discarded. This process ends when there is no cluster to be considered. Finally, the “response phases” are defined as the positions of the clusters remaining in ResponseSchedule $\mathbb {S}$.

### Determination of the number of clusters

The number of gene clusters was chosen empirically by examining how many ground truth genes were included in the clustering result. In our experiment, the top-ranked DEGs (i.e., top 10% DEGs among consensus DEGs in step 1) were selected as ground truth genes. Then, HTRgene was performed for the number of clusters, *K*, increased from 50 to half of the number of consensus DEGs by 50. Finally, K was selected to maximize the F1 score, which measures the association between the resultant genes and the top-ranked DEGs. The best K was 200 in both cold and heat experiments.

Alternatively, the user can use genes with stress-related Gene Ontology (GO) terms to determine the number of clusters. However, in this paper, genes with cold/heat stress related GO terms are used to evaluate the performance of tools in further analysis (“Performance comparison with existing methods” section). Thus, we used top-ranked DEGs rather than stress-related genes to evaluate the performance of the clustering algorithm without any prior knowledge.

## Results and Discussion

### HTRgene analysis of Heterogeneous time-series dataset of cold and heat stresses

HTRgene analysis was performed for heat and cold stress time-series data in Arabidopsis. Raw data of each stress were collected from GEO [25] and ArrayExpress [26]. This study focused on detecting genes and aligning them according to their response time to a single stress factor. Thus, the recovery phase data were excluded from the dataset. The collected raw data were processed and quantile normalized using the affy R package [20]. Tables 1 and 2 showed the heterogeneous meta-properties of 28 and 24 time-series sample datasets for cold and heat stress, respectively.

**Table 1:** Heterogeneous meta-properties of 28 time-series gene expression dataset for cold stress treatment

No.	Sample ID	Eco-type	Geno-type (NT: non-transgenic)	Age (days)	Tissue	Temperature (^∘^C)	Time points (minutes (m) or hours (h) after treatment)
1	E-MTAB-375	Columbia	NT	14	rosette leaf (low light)	4	0h, 5m, 10m, 20m, 40m, 1h, 80m, 100m, 2h, 140m, 160m, 3h, 200m, 220m, 4h, 260m, 280m, 5h, 320m, 340m, 6h, 10h40m, 21h20m
2	E-MTAB-375	Columbia	NT	14	rosette leaf (dark)	4	0h, 5m, 10m, 20m, 40m, 1h, 80m, 100m, 2h, 140m, 160m, 3h, 200m, 220m, 4h, 260m, 280m, 5h, 320m, 340m, 6h, 10h40m, 21h20m
3	GSE5621	Columbia	NT	14	shoot	4	0h, 30m, 1h, 3h, 6h, 12h, 24h
4	GSE5621	Columbia	NT	14	root	4	0h, 30m, 1h, 3h, 6h, 12h, 24h
5	GSE3326	Columbia	NT	14	seedlings	0	0h, 3h, 6h, 24h
6	GSE3326	Columbia	ice1	1	seedlings	0	0h, 3h, 6h, 24h
7	GSE55835	Columbia	NT	42	leaves	-3	0h, 8h, 24h, 72h
8	GSE55835	Rschew	NT	42	leaves	-3	0h, 8h, 24h, 72h
9	GSE55835	Tenela	NT	42	leaves	-3	0h, 8h, 24h, 72h
10	GSE5534	Columbia	NT	10	seedlings (plate)	4	0h, 1h, 24h, 168h
11	GSE5535	Columbia	NT	10	seedlings (soil)	4	0h, 1h, 24h, 168h
12	GSE53990	Columbia	NT	28	9-11th adult leaves	4	0h, 48h, 120h
13	GSE53990	Columbia	rcf	28	9-11th adult leaves	4	0h, 48h, 120h
14	GSE39090	Columbia	NT	14	seedlings	4	0h, 12h, 24h
15	GSE39090	Columbia	rcf	14	seedlings	4	0h, 12h, 24h
16	GSE37130	C24	NT	20	seedlings	4	0h, 3h, 24h
17	GSE37130	Columbia	NT	20	seedlings	4	0h, 24h
18	GSE43818	Columbia	NT	21	entire aerial part	4	0h, 24h
19	GSE43818	Columbia	camta1/2/3	21	entire aerial part	4	0h, 24h
20	GSE55906	WS-2	NT	11	entire aerial part	4	0h, 24h
21	GSE55906	WS-3	CBF2DN	11	entire aerial part	4	0h, 24h
22	GSE55907	Columbia	NT	12	seedlings	4	0h, 24h
23	GSE64575	Columbia	NT	10	entire aerial part	4	0h, 24h
24	E-MEXP-1345	Columbia	NT	45	leaf tip	4	0h, 24h
25	GSE19254	Columbia	NT	38	aerial tissues	4	0h, 48h
26	GSE19254	Columbia	sfr3	38	aerial tissues	4	0h, 48h
27	E-MEXP-3714	Columbia	NT	11	aerial tissues	1	0h, 2h
28	E-MEXP-3714	Columbia	ahk2ahk3	11	aerial tissues	1	0h, 2h

**Table 2:** Heterogeneous meta-properties of 24 time-series gene expression dataset for heat stress treatment

No.	Sample ID	Eco-type	Geno-type (NT: non- transgenic)	Age (days)	Tissue	Temperature (^∘^C)	Time points (minutes (m) or hours (h) after treatment)
1	E-MTAB-375	Columbia	NT	14	rosette leaf (normal light)	32	0h, 5m, 10m, 20m, 40m, 1h, 80m, 100m, 2h, 140m, 160m, 3h, 200m, 220m, 4h, 260m, 280m, 5h, 320m, 340m, 6h, 10h40m, 21h20m
2	E-MTAB-375	Columbia	NT	14	rosette leaf (dark)	32	0h, 5m, 10m, 20m, 40m, 1h, 80m, 100m, 2h, 140m, 160m, 3h, 200m, 220m, 4h, 260m, 280m, 5h, 320m, 340m, 6h, 10h40m, 21h20m
3	GSE5628	Columbia	NT	16	shoots	38	0h, 15m, 30m, 1h, 3h
4	GSE5628	Columbia	NT	16	roots	38	0h, 15m, 30m, 1h, 3h
5	GSE62163	Columbia	NT	21	shoots (EBR)	43	0h, 1h, 3h
6	GSE62163	Columbia	NT	21	shoots (no EBR)	43	0h, 1h, 3h
7	GSE63128	Columbia	NT	18	leaves	38	0h, 8h, 24h
8	E-MEXP-2760	Columbia	NT	35	shoot	40	0h, 20m, 1h
9	E-MEXP-2760	Columbia	mbf1c	35	shoot	40	0h, 20m, 1h
10	E-MEXP-3754	Columbia	NT	21	meristem	40	0h, 15m, 45m
11	GSE19603	Columbia	NT	56	above-ground	37	0h, 24h
12	GSE19603	Columbia	msh1/recA3	56	above-ground	37	0h, 24h
13	GSE43937	Columbia	NT	14	leaves	40	0h, 6h
14	GSE43937	Columbia	er-105	14	leaves	40	0h, 6h
15	E-MEXP-1725	Columbia	NT	35	leaves	37	0h, 2h
16	E-MEXP-1725	Columbia	hsf4-7	35	leaves	37	0h, 2h
17	GSE16222	Columbia	NT	4	seedlings	38	0h, 1h30m
18	GSE63372	Columbia	WT	7	seedlings	37	0h, 1h
19	GSE63372	Columbia	HSFA6b-OE	7	seedlings	37	0h, 1h
20	GSE63372	Columbia	HSFA6b-RD	7	seedlings	37	0h, 1h
21	GSE12619	Columbia	NT	7	seedlings	37	0h, 1h
22	GSE12619	Columbia	til1-1	7	seedlings	37	0h, 1h
23	GSE44053	Columbia	NT	7	seedlings	38	0h, 45m
24	GSE44053	Columbia	NT	7	seedlings	38	0h, 45m

**Fig. 2:**
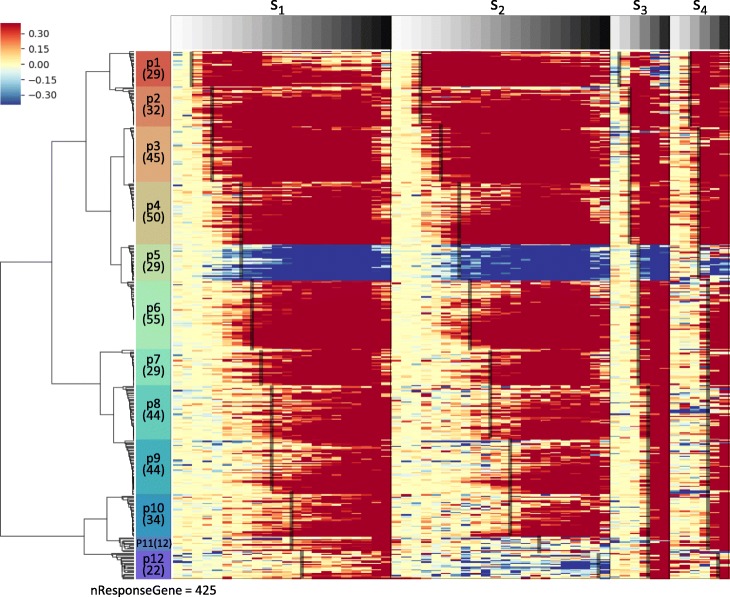
Heat map of a result of HTRgene analysis for cold stress data. The heat map includes 425 response order preserving DEGs that are grouped into 12 response phase clusters, which were discovered by HTRgene analysis of 28 cold stress time-series sample datasets. The rows of the heat map are 12 response phase gene clusters, and the numbers in parentheses are the number of genes for each cluster. The columns of the heat map are four time-series samples with more than five time points: *S*_1_ to *S*_4_. The red or blue color of the heat map indicates up or down change in gene expression level compared to the time point before stress (*T*=0). The black lines represent the response time point of a cluster in each sample. The heat map shows response times of gene clusters (the black line) defined by the HTRgene method are clearly propagated along the time axis in a conserved ordering across multiple samples

The HTRgene analysis outputted 425 and 272 candidate response genes that were assigned to 12 and 8 response phase gene clusters for cold and heat stress datasets, respectively. Figure 2 showed the heat map of 425 candidate genes to cold stress. It showed response times of gene clusters defined by the HTRgene method were clearly propagated along the time axis in a conserved ordering across multiple samples. In the next section, whether the response orders were consistent with actual stress signaling mechanisms or not were investigated through the literature review.

### Comparison with known cold stress pathway

**Fig. 3:**
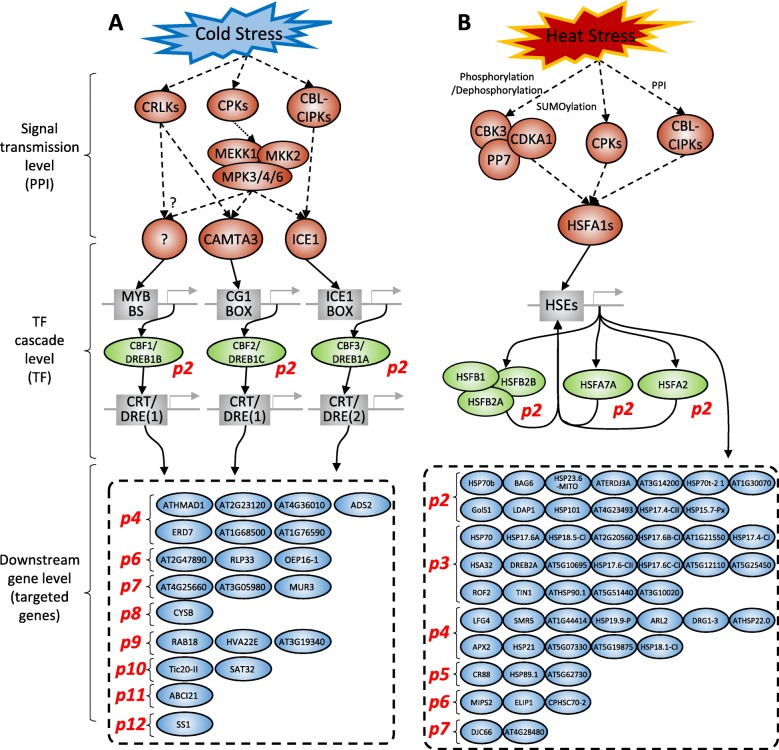
Comparison of HTRgene results to known biological pathways for cold and heat stress. The known cold and heat stress pathway are organized into signal transmission, TF cascade, and downstream gene level pathways. **a** Cold stress analysis. In the signal transmission level pathway, the cold stress signal sequentially activate stress response signaling proteins, such as CBL-CIPKs, CPKs, CLRK, MEKK1, MKK2, MPK3/4/6, CAMTA3, and ICE1 [27, 29]. In the TF cascade level pathway, CAMTA3 and ICE1 bind to MYB, CG1, and *ICE1*-box DNA cis-elements and initiate gene expression regulation of (DREB)/C-repeat binding factor (CBF) family including CBF1/DREB1B, CBF2/DREB1C, and CBF3/ DREB1A, respectively [28]. The HTRgene analysis result, CBFs that are known as “master switches” of the cold acclimation response [34] bind to CRT/DRE elements [35–37] and regulate many downstream genes that confer chilling and freezing tolerance to plants. The HTRgene analysis result included CBF1, CBF2, and CBF3 in the second response phase clusters “p2,” and the 21 donwstream genes of CBFs in the later phase clusters “p4,” “p6,” “p7,” “p8,” “p9,” “p10,” “p11,” and “p12.” **b** Heat stress analysis. In the signal transmission level pathway, the heat stress sequentially activates stress response signaling proteins, such as CBL-CIPKs, CPKs, PP7, CDKA1, CBK3, and HSFA1s [38]. In the heat stress TF cascade level pathway, HSFA1s that are the major regulators [45] of heat stress response initiate gene expression regulation of heat shock responsive TFs: HSFB1A, HSFB2A, HSFB2B, HSFA2, and HSFA7A, [38]. Then, transcriptional upregulation is accelerated in a feed-forward fashion that HSFBs, HSFA7A, and HSFA2 bind to *HSE* elements and up-regulate themselves again [46]. In the downstream level pathway, the heat shock factor TFs regulate the heat stress responsive downstream genes [47–49]. The HTRgene analysis assigned heat shock factors, HSFA2, HSFA7A, and HSFBs, to the second response phase “p2.” and the 52 downstream genes of the heat shock factors to the later response phases, “p2,” “p3,” “p4,” “p5,” “p6,” and “p7.”

The HTRgene analysis for cold stress data discovered 425 response order preserving DEGs belonging to 12 response phase clusters. The results were compared to known cold stress pathway genes summarized in review papers [27–29]. Figure 3a shows a three-level structure of the cold stress pathway: signal transmission, transcription factor (TF) cascade, and downstream gene level pathways.

The cold stress signal, in the signal transmission level pathway, affects membrane rigidity and changes the concentration level of Ca ^2+^. Then, the activation status of proteins are sequentially changed, such as CBL-CIPKs, CPKs, CLRK, MEKK1, MKK2, MPK3/4/6, CAMTA3, and ICE1 [27, 29]. HTRgene analysis did not include these genes as the result. We could biologically interpret why HTRgene analysis result did not include the signal transmission level pathway genes; the actions in the signal transmission level pathway, such as phosphorylation, ubiquitination, and SUMOylation [27–29], affect the proteins’ structures but not their expression levels.

CLRK is a Ca ^2+^/CaM-regulated receptor-like kinase that activates MEKK1-MKK2-MPK4/6 [30] and it could induce the expression of TFs such as MYB15 and ICE1 [31]. MEKK1 (MAP kinase kinase 1) activates MKK2 (Mitogen activated protein kinase kinase2) by phosphorylation and then MKK2 phosphorylates MPK4/6 under cold stress [32]. HOS1 (High Expression of Osmotically Responsive 1), an ubiquitin E3 ligase, reduces expression of ICE1 (Inducer of CBP Expression 1) target genes by ubiquitinating ICE1 [33], which is a basic helix-loop-helix transcription factor could regulate the expression of MYB15 and CBFs in low temperature signaling pathway [33].

CAMTA3 and ICE1 were activated genes at the last stage of the signal transmission level pathway. In the TF cascade level pathway, CAMTA3 and ICE1 bind to MYB, CG1, and *ICE1*-box DNA cis-elements and initiate gene expression regulation of (DREB)/C-repeat binding factor (CBF) family including CBF1/DREB1B, CBF2/DREB1C, and CBF3/ DREB1A, respectively [28]. CBFs are known as “master switches” of the cold acclimation response [34] because they regulate many downstream genes that confer chilling and freezing tolerance to plants by binding to CRT/DRE elements [35–37]. The HTRgene analysis result included CBF1, CBF2, and CBF3 in the second response phase clusters “p2”.

In the downstream gene level pathway, HTRgene assigned 21 genes that were reported as downstream genes of CBFs to the “p4,” “p6,” “p7,” “p8,” “p9,” “p10,” “p11,” and “p12” response phase gene clusters, which were later than the response phase of CBFs. Collectively, it was shown that the HTRgene analysis successfully reproduced known biological mechanisms for cold stress.

### Comparison with known heat stress pathway

The integrated analysis for heat stress data produced 272 candidate response genes in 7 response phase clusters. The results were also compared to the known heat stress pathway [38]. Figure 3b shows a three-level structure of the heat stress pathway: signal transmission, TF cascade, and downstream gene level pathways.

The heat stress signal, in the signal transmission level pathway, alters membrane rigidity and the concentration level of ROS and Ca ^2+^. Then, the activation status of some proteins are sequentially changed, such as CBL-CIPKs, CPKs, PP7, CDKA1, CBK3, and HSFA1s [38]. The HTRgene analysis result did not contain these genes. The result was possible because the signal transmission level pathway transmit the stress signal through the molecular actions, such as phosphorylation, dephosphorylation, SUMOylation, and protein–protein interaction [38], which do not change their gene expression levels but alter the proteins’ structures.

CBK3 is a well-known CaM-binding protein kinase that regulates phosphorylation of HSFA1 positively in heat-shock response [39]. PP7 (Protein phosphatase 7) acts as “calcineurin-like” phosphatase, interacting with CaM in plants. AtPP7 is also known as a phosphatase of HsfA1 in heat shock response and it is involved in crypto-chrome signaling [38, 40]. CDKA1 (Cyclin-Dependent Kinase A1) is one of the main kinases related to transition points in the cell cycle. It also phosphorylates HsfA1 and regulates the DNA binding ability of HsfA1 [38]. HSFA1s (Heat shock factor A1) is a major transcriptional regulator during heat stress and acts in other abiotic stress responses [41]. It has been reported that the reactive electrophile species (RES) oxylipins through the four master regulator transcription factors, HSFA1a, b, d, and e, that is essential for short-term adaptation to heat stress in Arabidopsis [42]. CPK (Calcium dependent protein kinase) is a Ser/Thr protein kinase that acts Ca ^2+^ sensing and kinase function involved in development and various abiotic stresses responses [43]. CBL-CIPKs builds a complex with Calcineurin B-like (CBL), a calcium-binding protein, and CBL-interacting protein kinases (CIPKs). This complex plays an important role in calcium signaling pathways during cold stress [44].

HSFA1s are the major regulators in the heat stress TF cascade level pathway [45]. However, they did not appear in the HTRgene analysis result. It is biologically interpretable since the molecular mechanisms that activate them are protein-structure modifying actions. HSFA1s that bind to *HSE* elements initiate gene expression regulation of heat shock responsive TFs: HSFB1A, HSFB2A, HSFB2B, HSFA2, and HSFA7A, [38]. Then, transcriptional upregulation is accelerated in a feed-forward fashion that HSFBs, HSFA7A, and HSFA2 bind to *HSE* elements and up-regulate themselves again [46]. Among the direct target TFs of HSFA1, HTRgene analysis assigned HSFA2, HSFA7A, and HSFBs to the second response phase “p2.”

Then, the heat shock factor TFs regulate the heat stress responsive downstream genes in the downstream level pathway [47–49]. Among the downstream genes, 52 genes were included in late response phase clusters, “p2,” “p3,” “p4,” “p5,” “p6,” and “p7.” Collectively, the agreement between HTRgene result and the known heat stress pathway showed that the HTRgene analysis successfully reproduced known biological mechanisms for heat stress.

### Enrichment analysis for clusters

**Fig. 4:**
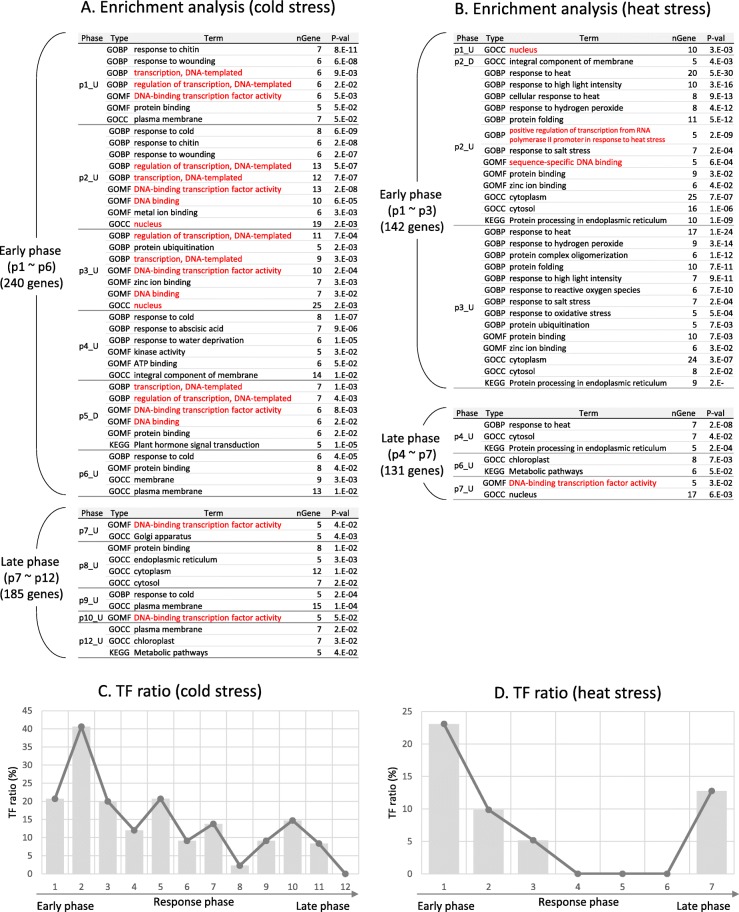
Enrichment analysis and TF ratio. Gene ontology (GO) and KEGG enrichment analyses were performed for cold (A) and heat (B) stress. There were 12 and 7 clusters for cold and heat stress, respectively. More GO and KEGG terms were enriched in six early phase clusters for cold stress and three early phase clusters for heat stress than in six later phase clusters for cold stress and four later phase clusters for heat stress. Functional terms related to transcription factors were enriched in early phase clusters. The terms are GO biological process term “transcription, DNA-templated”, the GO molecular function term “DNA-binding transcription factor activity”, and the GO cellular process term “nucleus”, which are highlighted by red color. We also examined how the proportion of TFs in the cluster changes as the response progresses for cold (C) and heat (D) stress. The result showed that the fraction of TFs was high in the early phase, while the TF fraction was decreased with the passage of time

GO term and Kyoto Encyclopedia of Genes and Genomes (KEGG) pathway enrichment analyses of 12 and 7 clusters for cold and heat stress, respectively, were performed for cold stress (Fig. 4a) and heat stress (Fig. 4b). More GO and KEGG terms were enriched in six clusters in the early phase for cold stress and three clusters in the early phase for heat stress. Functional terms related to transcription factors were enriched in early phase clusters. Many of nuclear targeting genes including TFs and genes with conserved DNA binding domains were present in p1 through p6, i.e., the early stage of signaling cascade, which could be defined as a cold signal reception stage. Additionally, genes coded for protein modifying kinase and genes involved in remodeling membrane properties were found in the early phases. In the late phases, however, many of the events happened outside the nucleus such as the micro-organelles such as Golgi, ER, chloroplast and plasma membrane. This tendency was observed in both cold and heat stress. We also examined how the proportion of TFs to genes in the cluster changes as the response progresses for cold (C) and heat (D) stress. The result showed that the fraction of TFs was high in the early phase as shown in Fig. 4c, while the TF fraction was decreased as the signal progresses as shown in Fig. 4d.

### Network analysis of clusters

**Fig. 5:**
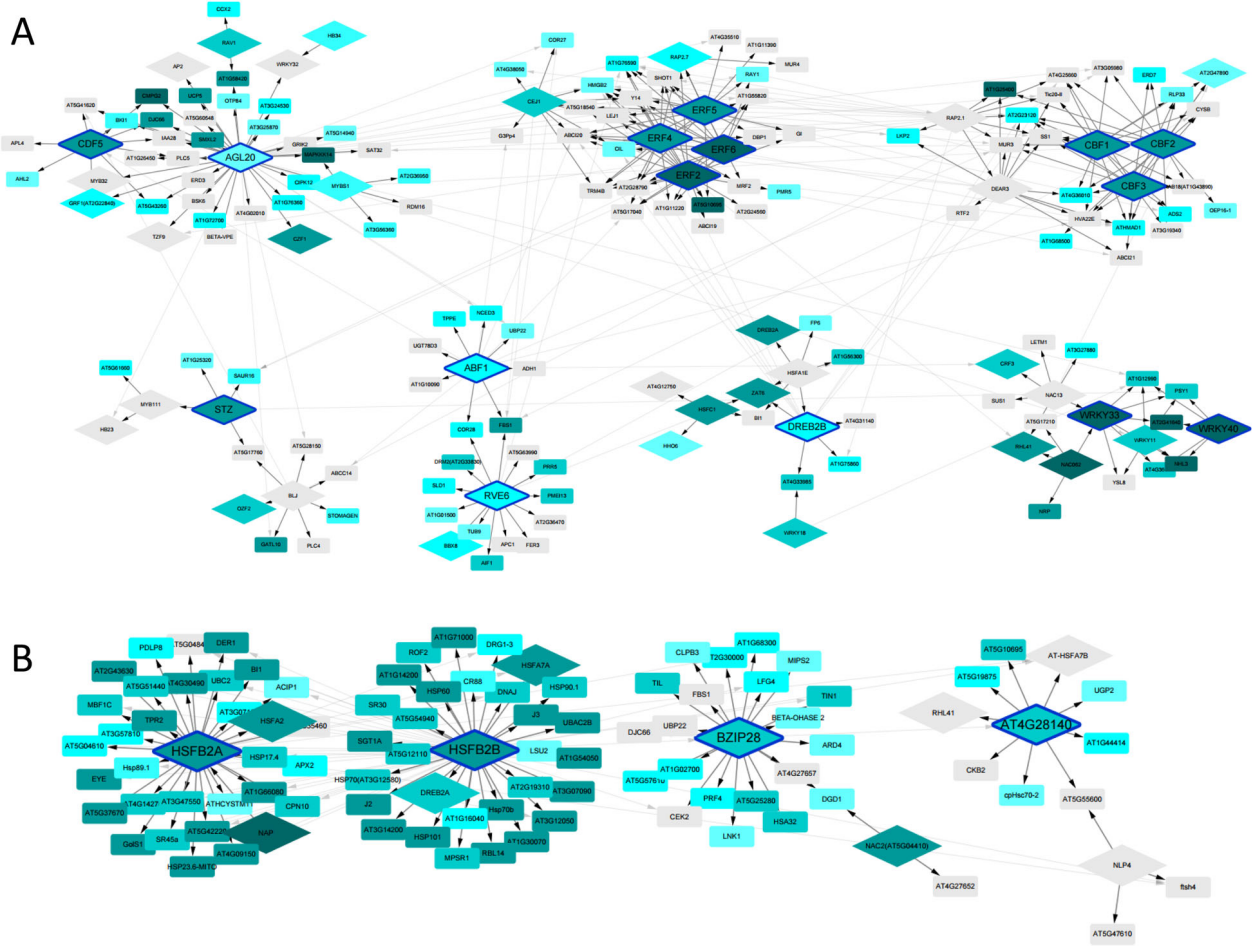
TF network analysis. The TF network analysis produced seven and four clusters for cold (**a**) and heat (**b**) stress, respectively. In the TF network clusters, hub regulator TFs that regulate over the target five genes (TG) of these clusters were observed, which are C1 (AGL, CDF5), C2 (ERF2, ERF4, ERF5, ERF6), C3 (CBF1, CBF2, CBF3), C4(STZ), C5(ABF1, RVE6), C6(DREB2B), and C7(WRKY33, WRKY40) for cold stress and C1(HSFB2A), C2(HSFB2B), C3(BZIP28), and C4(AT4G28140) for heat stress. The rhombus nodes represent TFs, and rectangular nodes represent TGs. The blue nodes represent early phase cluster genes and grey nodes late phase cluster genes. It shows that the hub regulator TFs of early half phase clusters regulate the TGs of late half phase clusters

We investigated how TFs are likely to regulate other genes through TF network analysis. To construct the TF network, a template TF network including 599 TF was downloaded from PlantRegMap database. The template TF network was refined by TF binding motif existence. Then, a network clustering algorithm, GLay [50] in the clusterMaker2 [51] package, was used to generate subnetwork clusters (Fig. 5). To identify important TF regulators, we compiled TFs, each of which has five or more target genes in one cluster. They are summarized as cluster-numbers(TFs): C1 (AGL, CDF5), C2 (ERF2, ERF4, ERF5, ERF6), C3 (CBF1, CBF2, CBF3), C4 (STZ), C5 (ABF1, RVE6), C6 (DREB2B), and C7 (WRKY33, WRKY40) for cold stress and C1 (HSFB2A), C2 (HSFB2B), C3 (BZIP28), and C4 (AT4G28140) for heat stress. Most of the important TF regulators were in the early phase clusters, and TGs of the TFs were present in the late half phase clusters. The network analysis suggests that stress response might start from hub TFs in early phases and propagates to TGs in downstream clusters and each downstream cluster regulates specific biological function.

### Performance comparison with existing methods

HTRgene was evaluated in comparison with existing tools. Qualitatively, HTRgene produces more informative output than other stress data analysis tools because it discovers not only candidate response order preserving DEGs but also response phases. However, DEG detection tools, e.g., DESeq [8], edgeR [9], and limma [10], generate DEGs only. Other pattern-based tools, such as ImpulseDE [14] also report differentially patterned genes between control and case time-series samples but do not provide response phases.

**Fig. 6:**
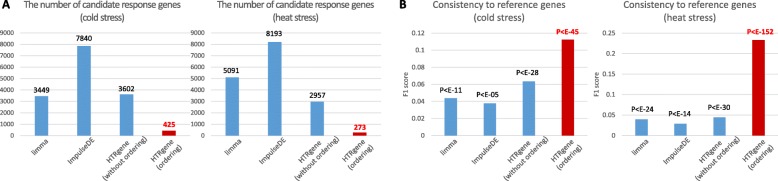
Performance comparison with existing tools. **a** The number of genes and **b** F1 scores for the results of limma, ImpulseDE, HTRgene without/with ordering analysis. F1 score is used to quantify the accuracy of method by comparing the resulting genes to ground truth genes that are labeled as the cold and heat stress related GO terms. Chi squared test is used to measure *p*-values

HTRgene was quantitatively compared with other tools in terms of accuracy of determining candidate stress response genes only because the existing tools do not provide response phases. First, we determined ground truth genes as 330 and 158 genes with GO annotation “response to cold” and “response to heat” from the TAIR database [19]. Then, the DEG detection tools, limma, ImpulseDE, were compared to HTRgene in terms of accuracy of discovering the ground truth genes. In addition, HTRgene without ordering and with ordering were considered separately in order to trace how much improvement was made by ordering of genes. Figure 6a showed the number of candidate response genes determined from the analysis of limma, ImpulseDE, HTRgene without ordering, and HTRgene with ordering; 3449, 7840, 3602, and 425 for cold stress analysis, and 5091, 8193, 2957, and 272 for heat stress analysis, respectively. Among the genes, 41, 56, 124, and 41 were ground truth genes for cold stress; and 73, 83, 69 and 49 ground truth genes for heat stress, respectively. Figure 6b showed F1 scores for the results of limma, ImpulseDE, HTRgene without/with ordering analysis. HTRgene provided the best F1 score over the other tools for both cold and heat stress analysis. The number of DEGs, precision, recall, F1 scores, and *p*-value of Chi squared test are summarized in Additional file 1: Table S1 and S2.

### Characteristics of HTRgene

To detect stress response signaling genes, HTRgene is developed to find a specific pattern, the ordering of response time of genes preserved among multiple gene expression time-series data. However, the problem of determining and ordering response time has a high complexity of *O*(*n*!), where *n* is the number of genes. We thus use clustering analysis to reduce the complexity of the problem from the number of genes to the number of gene clusters. Also, we take a greedy approach to find the longest ordering of response time. The greedy approach scans gene cluster by gene cluster starting from gene clusters of more differential expression. Thus, although our greedy-based method could not produce the globally optimal solution, the result of our approach is likely to include differentially expressed genes, which is a very clear signal of stress.

The results in “Performance comparison with existing methods” section shows the positive effect of ordering quantitatively. HTRgene methods with or without ordering produced about 3000 and 300 genes as the results. Measuring association between the results and known stress-related genes showed that ordering decreased recall about two-fold, but, increased precision over three-fold, resulting in the increment of F1 score and significance of Chi squared test. Collectively, these results showed that the ordering process of HTRgene improve DEG selection effectively by reducing the number of outputted DEGs and improving association with known stress genes (*p*<10^−45^).

Circadian rhythm is one of the factors that can affect the DEG result over time in plants. In general, circadian rhythm effects are differently measured in different time series datasets. Thus, when multiple time series datasets are integrated, circadian rhythm effects look like random noise, resulting in the exclusion of circadian rhythm-related genes in results. For example, circadian rhythm-related genes, such as, *ERD7*, *LKP2*, and *COR27*, were excluded after consideration of the response ordering. In addition, some experiments provide non-stress-treated time-series samples for control data (e.g., cold dataset 1 and 2 in Table 1). We think it would be a good future research to utilize these non-stress data.

## Conclusion

Measuring time series data is expensive, thus a computational method to integrate multiple heterogeneous time-series gene expression datasets is a very useful tool. However, there are several challenges for integrating time series datasets. The main challenge is that the datasets are heterogeneous in terms of the time-domain (the number of time points and intervals are different) and phenotype-domain (the tissue of samples and the age of samples are different).

We developed and implemented HTRgene, a method to integrate multiple heterogeneous time-series gene expression datasets to find the ordering of response time of genes that are commonly observed among multiple time-series samples. Our strategy of defining and using response times is very effective in producing not only gene clusters but also the order of gene clusters.

The utility of HTRgene was demonstrated in the investigation of stress response signaling mechanisms in Arabidopsis. The HTRgene integration analysis for 28 and 24 time-series sample gene expression datasets under cold and heat stress successfully reproduced known biological mechanisms of cold and heat stress in Arabidopsis.

## Supplementary information

**Additional file 1** **Table S1**. Association between predicted genes and ground truth genes for cold stress analysis. **Table S2**. Association between predicted genes and ground truth genes for heat stress analysis.

## Data Availability

The software package implementing the HTRgene algorithm and the multiple gene expression datasets for cold and heat stress used in this paper are available at http://biohealth.snu.ac.kr/software/HTRgene.

## Abbreviations

DEG: Differentially expressed gene
GO: Gene Ontology
KEGG: Kyoto encyclopedia of genes and genomes
RNA-Seq: RNA sequencing
RT: Response time
TF: Transcription factor
