# Supplementary material for: HTRgene: a computational method to perform the integrated analysis of multiple heterogeneous time-series data: case analysis of cold and heat stress response signaling genes in Arabidopsis
Source: BMC Bioinformatics. 2019 Dec 2;20(Suppl 16):588. doi: 10.1186/s12859-019-3072-2 (PMC6886170; doi:10.1186/s12859-019-3072-2)
Supplement: Supplementary file 1 — Additional file 1 Table S1. Association between predicted genes and ground truth genes for cold stress analysis. Table S2. Association between predicted genes and ground truth genes for heat stress analysis. [file 12859_2019_3072_MOESM1_ESM.docx]

**Supplementary Table S1.** Association between predicted genes and ground truth genes for cold stress analysis. The results (*A*) are compared with a set of ground truth genes (*B*) that are annotated with cold-stress-related GO terms; n(B)=330, ±SE. The scores in bold indicate the best performance.

| method | Predicted target genes (A) | True positives =n(A∩B) | False positives =n(A-B) | False negatives =n(B-A) | True negatives =21430 -n(A∪B) | Precision | Recall | F1 score | Chi-squared-test (-log10(pval)) |
| --- | --- | --- | --- | --- | --- | --- | --- | --- | --- |
| limma | 3449 ± 286 | 85 ± 6 | 3364 ± 280 | 219 ± 6 | 17762 ± 280 | 0.06 ± 0.014 | 0.28 ± 0.02 | 0.04 ± 0.002 | 11.44 ± 0.817 |
| ImpulseDE | 7840 ± 612 | 145 ± 8 | 7696 ± 604 | 159 ± 8 | 13430 ± 604 | 0.02 ± 0.001 | **0.48 ± 0.026** | 0.04 ± 0.001 | 5.85 ± 0.735 |
| HeteroTimeDEG (without ordering) | 3602 | 124 | 3478 | 180 | 17648 | 0.03 | 0.41 | 0.06 | 28.31 |
| HeteroTimeDEG (with ordering) | 425 | 41 | 384 | 263 | 20742 | **0.1** | 0.13 | **0.11** | **45.55** |

**Supplementary Table S2.** Association between predicted genes and ground truth genes for heat stress analysis. The results (*A*) are compared with a set of ground truth genes (*B*) that are annotated with heat-stress-related GO terms; n(B)=158, ±SE. The scores in bold indicate the best performance.

| method | Predicted target genes (A) | True positives =n(A∩B) | False positives =n(A-B) | False negatives =n(B-A) | True negatives =21430 -n(A∪B) | Precision | Recall | F1 score | Chi-squared-test (-log10(pval)) |
| --- | --- | --- | --- | --- | --- | --- | --- | --- | --- |
| limma | 5091 ± 360 | 70 ± 3 | 5021 ± 358 | 77 ± 3 | 16263 ± 358 | 0.02 ± 0.004 | 0.48 ± 0.019 | 0.04 ± 0.004 | 24.34 ± 4.427 |
| ImpulseDE | 8193 ± 873 | 90 ± 5 | 8103 ± 868 | 57 ± 5 | 13180 ± 868 | 0.01 ± 0.002 | **0.61 ± 0.031** | 0.03 ± 0.004 | 14.42 ± 3.252 |
| HeteroTimeDEG (without ordering) | 2957 | 69 | 2888 | 78 | 18395 | 0.02 | 0.47 | 0.04 | 30.24 |
| HeteroTimeDEG (with ordering) | 345 | 43 | 302 | 104 | 20981 | **0.12** | 0.29 | **0.17** | **152.77** |
